# HIV–Host Cell Interactions

**DOI:** 10.3390/cells12101351

**Published:** 2023-05-09

**Authors:** Sepiso K. Masenga, Bislom C. Mweene, Emmanuel Luwaya, Lweendo Muchaili, Makondo Chona, Annet Kirabo

**Affiliations:** 1 HAND Research Group, School of Medicine and Health Sciences, Mulungushi University, Livingstone Campus, Livingstone 10101, Zambia; bischarm1st@gmail.com (B.C.M.); luwayaemmanuel@gmail.com (E.L.); muchailil2@gmail.com (L.M.); makondomtj@gmail.com (M.C.); 2 Vanderbilt University Medical Center, Department of Medicine, Division of Clinical Pharmacology, Room 536 Robinson Research Building, Nashville, TN 37232-6602, USA

**Keywords:** HIV, AIDS, immunity, cells, T- cell exhaustion, CCR5, CXCR4, sex, dendritic cells, CD4^+^ T cells, T lymphocytes

## Abstract

The development of antiretroviral drugs (ARVs) was a great milestone in the management of HIV infection. ARVs suppress viral activity in the host cell, thus minimizing injury to the cells and prolonging life. However, an effective treatment has remained elusive for four decades due to the successful immune evasion mechanisms of the virus. A thorough understanding of the molecular interaction of HIV with the host cell is essential in the development of both preventive and curative therapies for HIV infection. This review highlights several inherent mechanisms of HIV that promote its survival and propagation, such as the targeting of CD4^+^ lymphocytes, the downregulation of MHC class I and II, antigenic variation and an envelope complex that minimizes antibody access, and how they collaboratively render the immune system unable to mount an effective response.

## 1. Introduction

The human immunodeficiency virus (HIV) is a Lentivirus belonging to the *retroviridae* family, responsible for the HIV/AIDS pandemic [1]. Although discovered and declared a pandemic in the 1980s, there is evidence that pre-epidemic strains of HIV existed as far back as the 1920s [2,3,4]. Based on genetic and antigenic variations, HIV is divided into two types: HIV-1 and HIV-2. HIV-1 is the most virulent and widespread [5,6]. HIV-1 is responsible for the global pandemic, whereas HIV-2 is mainly confined to West Africa [7,8]. 

HIV targets and infects Cluster of Differentiation 4-positive (CD4^+^) cells, predominately CD4^+^ T helper lymphocytes [9]. To mount a successful invasion, HIV requires the presence of the CD4 receptor and the C-C chemokine receptor type 5 (CCR5) or C-X-C chemokine receptor type 4 (CXCR4) co-receptor on the host cell [10,11]. Infection terminates in the death of the host cell; thus, infection invariably leads to the depletion of CD4^+^ T lymphocytes [12]. Since CD4^+^ T lymphocytes are the regulators of the adaptive immune system, their depletion effectively weakens the immune system, leading to the acquired immune deficiency syndrome (AIDS) stage of the infection [9]. Two biological phenotypes of HIV-1 exist, and these differ in terms of receptor tropism: X4 HIV-1 has an affinity for the CXCR4 receptor, whereas R5 HIV-1 has an affinity for the CCR5 receptors [13,14]. 

The most common mode of HIV transmission is unprotected sexual intercourse with an infected person [15,16]. Other modes of transmission include mother-to-child transmission, the use of contaminated needles and transfusion with infected blood [17,18,19,20,21]. Men who have sex with men and injectable drug users are at a higher risk of HIV infection [22,23,24,25]. Body fluids such as semen, vaginal fluids and blood of infected persons contain free-floating viruses and virus-infected CD4^+^-positive cells that facilitate the transmission of infection to the next cell or host [26,27,28,29].

An estimated 38.4 million people are living with HIV infection worldwide [30,31,32]. About 84.2 million have been infected, and 40.1 million have died since the beginning of the epidemic [30,31,32]. Sub-Saharan Africa has the highest prevalence of HIV infection, accounting for more than 70% of the global burden [33]. 

Tremendous progress in the understanding of the HIV molecular interaction with the host cell, the host cell responses to the virus and potential therapeutic implications of this interaction has been made since its discovery [34,35]. Vigorous research heralded the development of antiretroviral drugs a very important milestone in controlling the HIV pandemic [36]. Despite much progress in understanding the HIV–host cell interactions, the cure for HIV infection has remained elusive for four decades now [35,37]. There are still a lot of areas that we do not fully understand concerning the virus–host cell interaction mechanisms, which could be key to the development of novel therapeutic strategies for the cure of HIV infection [35,38]. 

The aim of our review, therefore, is to provide an in-depth overview of the current knowledge on the HIV life cycle and its multiple interactions with cellular proteins that promote or inhibit viral progression. The significance of such an undertaking is to highlight important interactions that help us to understand the pathogenesis of HIV. This information is important for future studies focused on targeted therapy.

## 2. Structure of HIV

The HIV-1 virion is spherical, with viral glycoprotein spikes (glycoprotein 120 (gp120) and gp41) that protrude from the viral envelope (env) [39]. The other structures include the Group antigens (Gag) responsible for directing the formation of virions from productively infected cells and the Pol protein containing enzymes critical for viral replication such as reverse transcriptase, protease and integrase [40,41]. The viral proteins such as viral protein R (vpr), viral protein U (vpu) and virion infectivity factor (Vif) are important for regulating nuclear import, replication, the degradation of CD4 molecules, virion release from cells and enhancing viral pathogenesis [42].

## 3. HIV Life Cycle

**Figure 1:**
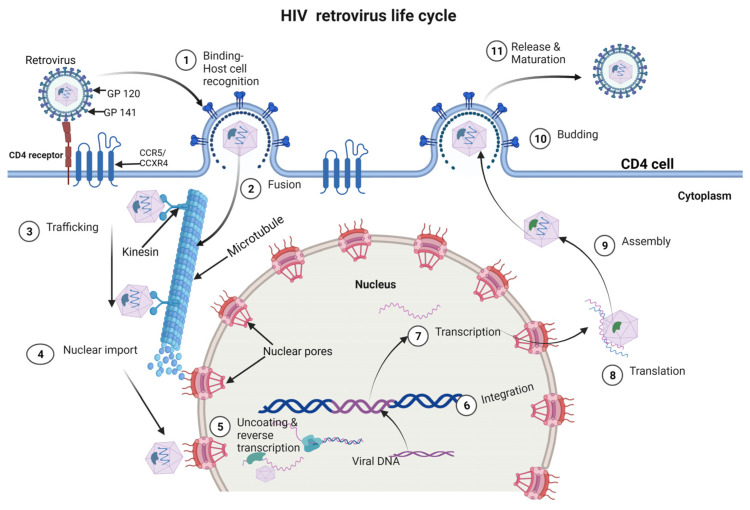
The life cycle of HIV-1.The early stage begins with virus interaction with the host cell receptors (1), which causes the virus to fuse and release its viral core into the host cell’s cytoplasm (2). Following this, the core is transported across the cytoplasm (3) as reverse transcription and nuclear import start to occur (4). The viral components are brought into the nucleus at the nuclear pore, where they are localized to transcriptionally active chromatin while uncoating and reverse transcription are carried out (5). Integration follows (6); then, viral genes are transcribed (7) and translated (8) into the Gag polyproteins, which assemble (9) and localize to the host membrane, followed by the occurrence of the budding of an immature virion (10). The viral protease cleaves the Gag polyprotein into its component, functional proteins during the last stage of the HIV-1 lifecycle, known as maturation (11).

The HIV life cycle consists of 11 phases and includes binding/attachment, fusion, trafficking, nuclear import, reverse transcription, integration, transcription/translation, assembly, budding and release [43,44] (Figure 1).

The initial step of HIV infection is the attachment of the virus to the CD4^+^ T cell receptor and co-receptor. The viral envelope glycoprotein, gp120, interacts with the CD4 receptor on the T cell surface, which triggers a conformational change in gp120, allowing it to bind to the co-receptor, either CXCR4 or CCR5 [45,46,47,48]. This binding leads to the exposure of the gp41 subunit, which mediates the fusion of the viral and cellular membranes [45,46,47,48]. After the viral envelope fuses with the host cell membrane, the core containing the viral genome and enzymes such as reverse transcriptase (RT), integrase (IN) and protease (PR) is released into the host cell cytoplasm and transported to the nucleus. The RT converts the viral RNA genome into double-stranded DNA (dsDNA), which is subsequently integrated into the host cell genome by the integrase enzyme with the aid of the Pre-integration Complex (PIC), a nucleoprotein complex comprising host and viral proteins, and the viral genome [49,50,51]. The integrated viral DNA is called a provirus, which remains dormant until activated by the host cell [52,53]. The provirus is transcribed by the host cell RNA polymerase II enzyme into messenger RNA (mRNA), which is then spliced and transported out of the nucleus into the cytoplasm [54,55]. The viral mRNA is translated into viral proteins by the host cell ribosomes. The Gag polyprotein is cleaved by the viral protease enzyme to form the structural proteins, including the matrix (MA), capsid (CA) and nucleocapsid (NC). The Gag-Pol polyprotein is also cleaved to produce the viral enzymes, including reverse transcriptase, integrase and protease [56,57]. The viral proteins and RNA genome are assembled into virions at the host cell membrane. The viral proteins, including Gag and Gag-Pol, bind to the viral RNA genome, forming the nucleocapsid core [35,58,59,60]. The core is surrounded by a lipid bilayer, which is derived from the host cell membrane and contains the viral envelope glycoproteins. Finally, the mature virions bud out of the host cell membrane, acquiring their envelope as they exit [35,58,59,60].

### HIV Receptors, Fusion and Uncoating Mechanism

Host cell receptors and co-receptors play a crucial role in determining the HIV tropism and pathogenesis. The role of the co-receptor of HIV is determined by the viral Env protein, and R5 strains are associated with early-stage disease, while X4 strains are associated with advanced disease [1,46,61,62,63]. Viral fusion and entry mechanisms are crucial for enabling the virus to infect host cells. Viral fusion proteins undergo significant conformational changes during the fusion process, overcoming energy barriers to fuse the membranes of the host cell and the virus [64,65,66]. Some viruses can also mediate cell–cell fusion, resulting in the creation of multinucleated cells that express significant amounts of viral antigens [67]. This process is dependent on the cellular environment and tissue structure. In order to develop effective vaccines and antibody-based treatments and minimize the spread of pathogenic viral variations, it is imperative to comprehend the mechanisms of viral fusion and entry [68]. 

The HIV capsid uncoats in the nucleus, and this contradicts a long-held theory that reverse transcriptase transforms HIV RNA to DNA in the cytoplasm [69]. Before viral genomic DNA is integrated into the host chromosomes, the coating must be removed [69,70]. The capsid interacts with nucleoporin 153 (NUP153), an essential component of nuclear pore complexes (NPCs) in vertebrates, and is required for the anchoring of NPCs. It also acts as the docking site of an importing karyopherin, and its phenylalanine-glycine motifs engage a common binding site on the HIV-1 capsid protein (CA) to facilitate the nuclear import of the viral nucleoprotein complex [71,72,73]. Nucleoporin 358 (NUP358) is a nuclear pore complex protein that is involved in the nuclear import of HIV-1 pre-integration complexes (PICs) [74]. Transportin-3 (TNP03) is a karyopherin that is involved in the nuclear import of HIV-1 PICs [75]. Cleavage and Polyadenylation Specificity Factor 6 (CPSF-6) is a cellular protein involved in pre-mRNA 3′ end processing that binds the HIV-1 capsid protein (CA) to facilitate the nuclear import of the viral nucleoprotein complex [76]. These molecules allow the viral complex to be imported and localized in the nucleus [71].

## 4. HIV-Related Factors Promoting Infection and Immune Evasion

The ability of HIV to evade the immune system is the core reason it has remained without a cure for decades [77,78]. HIV has various inherent mechanisms designed to escape or uncouple the host immune system [79]. 

HIV infection is characterized by progressive immune system impairment, leading to opportunistic infections, autoimmune diseases and malignancies [80]. The virus targets CD4^+^ T cells, macrophages and dendritic cells, leading to their depletion and dysfunction, with a CD4^+^ T cell count below 200 cells/mm^3^ considered diagnostic of AIDS [80,81,82]. The virus evades the immune system through several mechanisms, with the major ones being the downregulation of major histocompatibility complex (MHC) class I and II molecules, non-neutralizing antibody production and immune exhaustion [80]. Understanding the factors promoting HIV infection and immune evasion is crucial for the development of effective therapies and vaccines [83,84]. HIV can persist and evade the immune system due to several factors discussed below.

### 4.1. Downregulation of MHC Class I and II

HIV can evade the host immune system by downregulating the expression of MHC class I and II molecules, which are proteins that are essential for antigen presentation and recognition by immune cells [85,86]. This occurs at a molecular level through several mechanisms, including the ability of HIV to interfere with the transcription and translation of MHC class I and II genes, which reduces the overall expression of these molecules on the surface of infected cells [87,88]. This effect is mainly mediated by the HIV-1 accessory protein Nef [89], which is essential for viral pathogenesis and hence a potential target for antiretroviral drug discovery [42]. 

Nef interacts with the cytoplasmic tail of MHC I and II molecules and redirects them to the endocytic pathway for degradation [90,91]. One proposed mechanism for the HIV-1 Nef-mediated downregulation of cell surface MHC-I molecules is that Nef and Phosphofurin Acidic Cluster Sorting Protein 1 (PACS-1) combine to usurp the ADP ribosylation factor 6 (ARF6) endocytic pathway by a phosphatidylinositol-3 kinase (PI3K)-dependent process and downregulate the cell surface MHC-I to the trans-Golgi network [92].

The HIV Vpu protein also facilitates the degradation of MHC class I molecules. Vpu targets MHC I molecules for degradation by interacting with the host protein beta-TrCP, which recruits the E3 ubiquitin ligase complex to tag MHC I for degradation in the proteasome. The Vpu protein [93,94] interferes with the transport of newly synthesized MHC I molecules to the cell surface, where they are required for recognition by immune cells [83,95,96] by sequestering MHC-I intracellularly in the early stages of endocytosis and recycling [97]. Vpu interferes with the transport of newly synthesized MHC I molecules from the endoplasmic reticulum (ER) to the cell surface by targeting the host protein, tetherin (also known as BST-2 or CD317) [98]. Tetherin is a membrane protein that inhibits the release of virus particles from infected cells, thus limiting the spread of the virus. Vpu counteracts this antiviral mechanism by binding to tetherin and promoting its degradation through the proteasome pathway [99]. By degrading tetherin, Vpu enhances the release of viral particles from infected cells [100,101]. However, recent studies have shown that tetherin also plays a role in the transport of MHC I molecules to the cell surface [102]. Tetherin interacts with newly synthesized MHC I molecules and promotes their transport to the cell surface, where they can present viral antigens to the immune system [103]. Therefore, by targeting tetherin for degradation, Vpu impairs the transport of newly synthesized MHC I molecules from the ER to the cell surface, leading to a reduction in MHC I expression on the cell surface [104,105]. This impairs the presentation of viral antigens to cytotoxic T cells and thus helps the virus to evade immune surveillance [101]. 

As a result, the ability of the immune system to recognize and respond to HIV-infected cells is compromised, which allows the virus to evade immune clearance and persist in the host [83,95,96]. 

### 4.2. Production of Non-Neutralizing Antibodies

The viral envelope glycoprotein, gp120, is highly variable, and it can quickly mutate to escape recognition by neutralizing antibodies that target specific regions of the protein, thus leading to the production of non-neutralizing antibodies that can bind to gp120 but are unable to block virus entry [11,46].

Non-neutralizing antibodies can still play a role in HIV immune evasion. By binding to gp120, they can prevent the recognition of viral epitopes by neutralizing antibodies or T cells, effectively shielding the virus from immune surveillance [106]. Non-neutralizing antibodies can also trigger Fc receptor-mediated signaling, which can downregulate immune effector cells, such as Natural Killer (NK) cells and macrophages, leading to decreased antibody-dependent cellular cytotoxicity (ADCC) and phagocytosis of infected cells [107,108]. In addition, HIV can also use the immune system to its advantage by inducing polyclonal B-cell activation, which leads to the production of non-neutralizing antibodies that can distract the immune system and further facilitate viral evasion [109,110]. Overall, the production of non-neutralizing antibodies by HIV contributes to its ability to persist and evade the host immune system by several mechanisms, including blocking neutralizing antibody recognition, downregulating immune effector cells and inducing polyclonal B-cell activation [109,110].

### 4.3. Induction of Immune Exhaustion

HIV can induce immune exhaustion, which is a state of functional impairment of T cells, at a molecular level by several mechanisms. First, persistent antigen stimulation caused by HIV infection leads to T cell activation and proliferation, eventually leading to T cell exhaustion [12]. Second, HIV upregulates inhibitory receptors on T cells, such as programmed cell death receptor 1 (PD-1), cytotoxic T lymphocyte antigen-4 (CTLA-4) and T-cell immunoglobulin domain- and mucin domain-containing protein 3 (TIM-3), which negatively regulate T cell activation and function [12]. Third, HIV downregulates the expression of key transcription factors and cytokines, such as the T-Box protein expressed in T cells (T-bet), interferon-gamma (IFN-γ) and IL-2, that are necessary for effector T cell function [12,111]. These mechanisms collectively contribute to the development of T-cell exhaustion, leading to decreased immune surveillance and clearance of HIV-infected cells and ultimately allowing the virus to persist in the host [112].

### 4.4. Destruction of Virus-Specific T Helper Cells

HIV can evade the host immune system by destroying virus-specific T helper cells, which are important for coordinating the immune response against the virus [79]. This occurs at a molecular level through several mechanisms [113]. First, HIV can directly kill infected T helper cells by inducing apoptosis or programmed cell death [114]. Second, HIV-infected cells can also cause the bystander killing of uninfected T helper cells through the release of viral proteins, such as Tat, Nef and gp120, which activate apoptosis pathways in nearby cells through several mechanisms such as the upregulation of Fas, FasL and TNFα expression [115], the reduced expression of Bcl-2 and the activation of p53 [116]. Third, HIV proteins can induce cell death pathways by disrupting the normal functioning of cellular proteins and organelles, such as the mitochondria, which can lead to the death of infected and uninfected T helper cells [79,117,118]. The loss of T helper cells impairs the ability of the immune system to mount an effective response against HIV, allowing the virus to persist and replicate in the host [81].

### 4.5. The Emergence of Antigenic Escape Variants 

HIV can evade the host immune system through the emergence of antigenic escape variants, which are viral strains that have mutations in the viral proteins that are recognized by the immune system [119]. Several mechanisms favor this. First, HIV replicates at a high rate, which results in the generation of a large number of viral particles that can potentially acquire mutations [120]. Second, the HIV reverse transcriptase, the enzyme responsible for copying the viral genome, is highly error-prone, which increases the likelihood of mutations occurring during replication [121]. Third, the immune system exerts selective pressure on HIV by targeting specific viral proteins, which can result in the emergence of variants that are less recognizable by the immune system [79]. As a result, HIV can accumulate mutations that allow it to evade immune recognition and continue to replicate. This results in a diverse population of HIV variants that can persist in the host and avoid immune clearance [119,122].

### 4.6. Expression of an Envelope Complex That Minimizes Antibody Access

HIV can evade the host immune system by expressing an envelope complex that minimizes antibody access, which refers to the outer surface of the virus that is recognized by the immune system [123]. The envelope protein of HIV undergoes molecular-level changes through various mechanisms. The envelope protein of HIV is covered in sugar molecules, making it highly glycosylated, and this can prevent antibodies from binding to and neutralizing the virus by shielding vulnerable regions of the envelope protein from antibody recognition [124]. Another mechanism of evasion is that HIV undergoes rapid mutation of the envelope protein, which allows the virus to constantly evade antibody recognition [122,125]. By frequently changing the shape of the envelope protein, HIV can avoid recognition by antibodies that were produced against previous strains of the virus. This makes it difficult for the immune system to produce effective antibodies against HIV, which contributes to the ability of the virus to persist in the host [124].

### 4.7. Dysregulation of the JAK/STAT Pathway

Interferons are primarily produced and released by host cells such as immune cells (macrophages, dendritic cells, T cells) and non-immune cells (fibroblasts, epithelial cells) in response to viral infections, certain bacterial infections or other immune triggers [126]. Upon detecting viral particles, the host production of interferons creates an antiviral atmosphere which suppresses viral replication through the mechanism of inducing the expression of antiviral proteins and activating immune cells [127,128,129,130]. However, the virus can uncouple this host defense mechanism [131] by blocking the Janus kinase (JAK)/signal transducer and activator of the transcription (STAT) pathway, resulting in the termination of interferon production [132,133]. The JAK/STAT pathway plays a critical role in regulating inflammation during viral infections such as HIV [134]. Activators of the pathway could potentially help control HIV, but HIV Vpu and Nef disrupt the activation of JAK/STAT by IFN-α stimulation, reducing its induction [132]. HIV also promotes the degradation of Type 1 IFN JAK/STAT pathway components, suppressing the induction of specific Interferon-stimulated genes (ISGs) [135]. Various viruses have mechanisms for evading JAK/STAT signaling, and abnormal JAK/STAT signaling is associated with immune system dysregulation [83]. 

### 4.8. Other Factors That Promote HIV Infection 

The HIV-1 viral infectivity factor (Vif) is a 23-kDa protein found within the HIV-1 virion that plays a crucial role in the survival/invasion of host tissue by HIV [136]. It counteracts the APOBEC3 family of proteins, which are host cellular defense mechanisms that can mutate the genetic material of viruses, including HIV. Vif targets Apolipoprotein B mRNA Editing Catalytic Polypeptide-like (APOBEC3) proteins for degradation, allowing the virus to continue to replicate and spread [137]. Without Vif, HIV is much less able to infect and replicate in host cells [137]. Additionally, the Elongin–Cullin–SOCS (ECS) box site is involved in several HIV-related factors that promote infection and immune evasion [138,139]. The HIV-1 Vif can interact with the ECS box site on SOCS proteins, leading to the dysregulation of cytokine signaling pathways and promoting viral replication and immune evasion [138,139]. The ECS box site is also involved in the regulation of interferon signaling pathways, and the dysregulation of these pathways by HIV can contribute to immune evasion and pathogenesis of the virus [138,140,141]. Overall, the ECS box site plays a critical role in the regulation of immune responses, and understanding its role in HIV infection is important for developing new therapies for the virus [138,140,141]. 

Restriction by S-adenosylmethionine-dependent demethylase 1 (SAMHD1) limits the cGAS/STING-dependent innate and adaptive immune responses to HIV-1, and the Cyclic GMP-AMP synthase (cGAS) and the stimulator of interferon genes (STING) are involved in these responses [133]. SAMDH1 is a restriction factor for HIV-1 infection that has been shown to prevent innate sensing of infection via cGAS/STING, subsequently limiting innate and adaptive responses [142]. Furthermore, a class of NOD-like receptors (NLR), which are primarily associated with the inflammasome, have recently been associated with the production of interferons, which are significant in the fight against viral infections [143]. The NLRX1 subtype of NLR has been reported to appropriate STING to negatively regulate the interferon response, facilitating the replication of HIV-1 and DNA viruses [143]. 

## 5. Host Cell Mechanisms That Control Infection and Replication

HIV-1 infection progression is determined by both the virus and the host cells, with pattern recognition receptors (PRRs) playing a vital role in initiating the host immune response [144]. Early HIV-1 infection, the first hours to days after infection, in which the virus replicates in the cells such as the dendritic cells and macrophages/monocytes and is not detectable in the blood, is referred to as the “eclipse phase” [145]. The characteristics of early/recent infection include a high viral load and immune cell depletion. This eventually leads to immunodeficiency, and without treatment, individuals die of AIDS [146]. However, at the onset of infection, innate immune cells such as dendritic cells, NK cells, NKT cells, ϒδ T cells and B1 cells macrophage/monocytes respond to infection and also induce the cells of the adaptive immune system, the CD4^+^ and CD8^+^ T lymphocytes [147]. The innate immune response, requiring no gene rearrangement, is non-specific and uses pattern recognition receptors to recognize the HIV infection and induce other innate related factors against HIV [148]. These innate immune components include skin mucosal epithelial cells, phagocytes and NK cells, as well as a series of soluble factors, such as cytokines, chemokine and small molecular substances, such as complement and mannose-binding lectin [149]. The mechanism of response involving innate immunity is described as follows: dendritic cells recognize HIV through PRR such as the RIG-I-like receptors (RLR) including the retinoic acid-inducible gene 1 (RIG-1) and melanoma differentiation-associated gene 5 (MDA57), as well as the Toll-like receptors (TLRs) [150,151]. When activated, the RIG-1 and MDA5 trigger a downstream adaptor, the mitochondrial antiviral protein (MAVS), which further recruits another adaptor molecule, tumor necrosis factor-receptor associated factor 3 (TRAF3) [152]. The TRAF3 facilitates the recruitment of serine/threonine-protein TANK-binding kinase 1 (TBK1) and IϏβ kinase ε, leading to the phosphorylation and nucleus translocation of the transcription factor IFN-regulatory factor 3 (IFR3), which induces the expression and secretion of IFNs [153]. Cytokines are also produced when MAVS recruit IϏϏ-related kinases (IϏϏα, IϏϏβ and IϏϏϒ) and activate the NF-Ϗβ pathway by the phosphorylation and translocation of p65 [154]. 

The secreted IFNs produce an antiviral effect by autocrine and paracrine ligation to interferon-alpha/beta receptors (IFNAR) on cell surfaces [155]. This activates the downstream JaK/STAT signaling pathway through receptor-associated Jak1/TyK2 (tyrosine kinase) [156]. The phosphorylated STAT1 and STAT2 then form a heterodimer that interacts with IFN-regulatory factor 9 (IFR9) to form an IFN-stimulated gene factor 3 (ISGF3) transcription complex. ISGF3 translocate to the nucleus, where it binds to IFN-stimulated response elements (ISREs) in gene promoters, leading to the expression of IFN-stimulated genes to establish the host antiviral status that impairs viral replication and promotes the maturation of dendritic cells, promoting the activation of adaptive immune response [154]. 

Sentinel dendritic cells and macrophages are powerful, professional antigen-presenting cells that not only play a significant role in the initial response to infection but also activate adaptive immunity [149]. While sentinel dendritic cells are the first cells in response to infection, macrophages are the main effector cells involved in the late innate immune response and support the recruitment of inflammatory cells by secreting cytokines such as IL-1 and TNF-α [157]. Other cytokines, such as IFN-α and IL-15, which are secreted by dendritic cells and monocytes are significant in the activation of NK cells [158].

### 5.1. Pathogen Recognition Receptors (PRRs)

Pathogen Recognition Receptors (PRRs) are immune receptors that recognize conserved molecular patterns on pathogens, such as bacteria or viruses [159]. PRRs are differentially expressed by various immune cells including macrophages and dendritic cells [160]. PRRs are an essential component of the innate immune system and initiate downstream signaling that leads to the production of cytokines, chemokines and molecules capable of activating the adaptive immune response [161]. 

**Figure 2:**
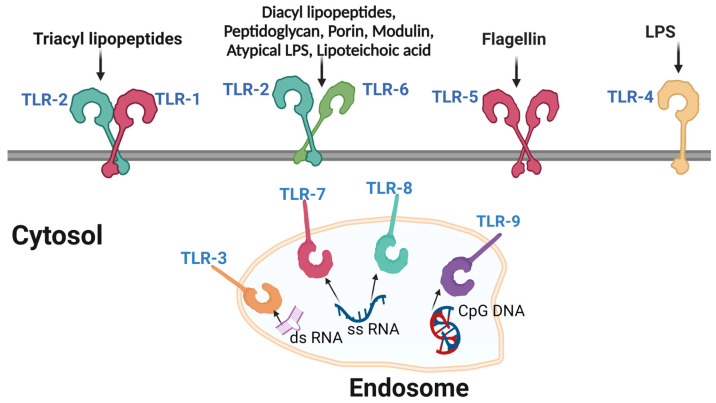
Toll-like receptors. Toll-like receptors act as antigen sensors for the immune system. They recognize a wide range of foreign antigens and thus help the immune system mount an appropriate response.

PRRs can be divided into several classes, such as Toll-like receptors (TLRs), Nod-like receptors (NLRs), RIG-I-like receptors (RLRs) and C-type lectin receptors (CLRs), among others [162]. TLRs (Figure 2) are the most studied and characterized PRRs and recognize a broad range of pathogen-associated molecular patterns (PAMPs), including lipopolysaccharides, lipoproteins and viral nucleic acids [163]. Once bound to their specific ligands, TLRs activate multiple downstream signaling pathways, including the NF-kB pathway and the interferon regulatory factor (IRF) pathway, leading to the expression of genes that drive the inflammatory and antiviral immune response [164,165].

In HIV infection, PRRs play a key role in recognizing the virus and initiating early immune responses [151]. HIV is quickly recognized by several PRRs, which leads to the production of cytokines, chemokines and type I IFN that stimulate immune cells, create an antiviral atmosphere and activate the adaptive immune response [166]. For example, TLR7 and TLR8, expressed by plasmacytoid dendritic cells (pDCs), recognize the single-stranded RNA of HIV, which triggers an antiviral cytokine response containing interferon-alpha (IFN-α) [167]. 

However, HIV has inherent capabilities of evading PRR recognition and establishing a persistent infection through the use of viral proteins such as Nef and Vpr, which can inhibit PRR signaling by downregulating PRR expression or blocking downstream signaling pathways [168]. 

### 5.2. Dendritic Cells

Dendritic cells (DCs) are among the first cells that encounter HIV, and being antigen-presenting cells, they are significant in the fight against the virus and the stimulation of the adaptive response [147,169]. While patrolling in the tissue, they can recognize antigens, process them and present them to T cells in secondary lymphoid organs, thereby activating the adaptive immune system. DCs can also secrete several diverse cytokines intended to upregulate the immune response by the secretion of cytokines; the exact type of cytokines secreted is dependent on the DC cell subtype and specific stimuli [148,170]. 

DCs are divided into three subtypes characterized by specific functions and markers, namely, plasmacytoid DCs (pDCs) and two subtypes of “classical” or “conventional” DCs (cDCs), cDC1 and cDC2 [171]. However, all DCs express CD4, a receptor involved in the binding and entry of HIV, together with its co-receptors, CCR5 and CXCR4 [157]. In addition to this, they also express CD83, a maturation marker, and CD80/CD86, which are activation markers involved in the antigen presentation and T-cell activation [147,149,157].

#### 5.2.1. Plasmacytoid Dendritic Cells (pDCs)

The most characteristic feature of the pDCs is the production of the type 1 interferon that promotes a strong antiviral immune response [171]. Plasmacytoid DC expresses TLR7, which enables it to recognize the virus after uptake by endocytosis and activate a signaling cascade that leads to the maturation of pDCs, the production of IFN-α, IFN-β and TNF-α and the expression of chemokine receptors such as the CCR5, CD40, CD80 and CD86 co-stimulatory molecules [148]. In addition to the expression of chemokine receptors, type I interferons also promote the production of proteins, cell growth and survival to establish an “antiviral state” [148,170]. When TLR7 recognizes the viral nucleic acid, it leads to the recruitment of the cytosolic Toll-interleukin-1 receptor (TIR)-containing adaptor MyD88, which is used by most TLRs [149]. The MyD88 forms a complex with members of the IL-1 receptor-associated kinase (IRAK) family and recruits the Interferon Regulatory Factor 7 (IRF7) [149]. When IRF7 is phosphorylated by IRAK1 and translocated to the nucleus, it regulates the expression of the type 1 interferon [149].

#### 5.2.2. Conventional Dendritic Cells (cDCs)

Conventional DCs function mainly as specialized APCs; however, they also produce several cytokines upon recognition of an antigen, mainly inflammatory cytokines including IL-6, IL-12, IL-15, IL-23, TNF and IL-1β all, of which are significant in restraining HIV-1 infection as compared to pDCs, which are known for the secretion of a large amount of type I interferons [147]. Conventional DCs are important in bridging innate immunity and adaptive immunity by presenting antigens to T cells [147]. Whereas cDCs1 are distinguished by their effective MHC class I-mediated priming of CD8^+^ T cells, cDC2 have a broad variety of factors generated and high cross-presenting abilities, promoting a potent activation of T_h_1, T_h_2 and T_h_17 as well as CD8^+^ T cell responses [147,149,157,170].

While DCs play a key role in mounting a strong immune response, they are widely thought to contribute to the immune exhaustion seen in chronic HIV infection through several mechanisms [172]. In acute HIV infection, DCs are activated and produce high levels of type I IFNs, which are key in viral control [157,173]. However, the persistent stimulation of DCs to secrete IFN 1 seen in HIV infection leads to the continuous activation of the innate and adaptive immune system; furthermore, IFN 1 has direct pro-apoptotic activity and upregulates TNF-related apoptosis-inducing ligand (TRAIL) on CD4^+^ T cells [174]. The upregulation of TRAIL in DCs during chronic HIV infection promotes the apoptosis of uninfected CD4^+^ T cells via the NF-kB pathway activation [175]. Some TLR receptors, including TLR 7, which are usually elevated in chronic HIV infection, activate the NF-kB pathway in DCs and induce TRAIL expression [176]. Additionally, the upregulation of TRAIL expression can also be mediated by the IFN-1: binding of TNF-1 to its receptor TNFR-1, and DCs can activate the JAK/STAT pathway, which can in turn upregulate TRAIL expression [177,178]. Second, DCs may also contribute to immune exhaustion by inducing regulatory T cells (Tregs): Tregs suppress the functions of many immune cells including T cells and dendritic cells [179]. In HIV infection, DCs have been shown to induce the differentiation and expansion of Tregs by the activity of the immunoregulatory enzyme, indoleamine 2,3 dioxygenase (IDO), which they express [172,180]. IDO breaks down tryptophan to kynurenine, a factor required for the conversion of inflammatory T cells to Tregs; thus, IDO is important in the expansion of Tregs in chronic HIV [181].

### 5.3. Macrophages

Macrophages are key players in innate immune responses to pathogens, and their ability to destroy a wide range of pathogens while doubling as APCs makes them a vital component of the innate immune system [182]. Unlike most cells of the myeloid lineage, macrophages have a longer life span, ranging from months to years [183]. Macrophages are widely distributed in the body and reside in almost every tissue of the body [184]. While initially thought to be incapable of self-renewal, there is evidence that tissue macrophages can and do replenish themselves [185,186,187].

Viral interaction with macrophages is very important in the HIV disease course [188]. In the sexual transmission of HIV, macrophages encounter HIV in the genital mucosa along with CD4^+^ T cells and DCs [189,190]. Macrophages play a crucial role in the immune response to HIV infection in the early stages of the disease, as their primary function is to engulf and clear viral particles and infected cells [191,192]. 

One pathway that plays a critical role in macrophage immune responses during early infection is the Toll-like receptor (TLR) pathway [193,194]. Macrophages express several TLRs, and upon the binding of PAMPs to the TLRs, several signaling pathways are initiated, such as the activation of NF-κB, and interferon regulatory factors (IRFs) [163,195]. These transcription factors lead to the production of cytokines, chemokines and type I interferons (IFNs), which can inhibit viral replication and activate the adaptive immune response [129]. 

Macrophages also use the inflammasome pathway to destroy the virus in acute infection: the inflammasome is a cytosolic multimolecular complex that serves as a platform for the activation of caspase-1 and the processing and release of pro-inflammatory cytokines such as IL-1β and IL-18 [196]. Macrophages activate the inflammasome following the recognition of PAMPs or damage-associated molecular patterns (DAMPs) released by infected cells [197]

Additionally, autophagy in macrophages also plays a crucial role in controlling HIV replication. Autophagy is a cellular process that is involved in the degradation of cytoplasmic contents, including viral particles [198]. In macrophages, HIV can be sequestered within the autophagosome and subsequently degraded in the lysosome [199]. However, HIV can evade this mechanism and promote its replication within the macrophage by hijacking the autophagy machinery and continue surviving and replicate in macrophages [200]. 

Like the CD4^+^ T cells, macrophages possess the CD4 receptor and the receptors CCR5 and CXCR4, thus making them equally susceptible to HIV infection [201]. Macrophages are particularly susceptible to R5 viruses, and coincidentally, most sexually transmitted HIV viruses are R5 [202]. Macrophages are believed to play a vital role in HIV latency and cell-to-cell viral spread, even in persons on antiretroviral drugs for several reasons [77,203]. It has been shown that HIV-infected macrophages have longer and more stable interactions with CD4^+^ T cells compared to uninfected macrophages, implying that some viral mechanisms enhance their T cell interactions, thus aiding viral spread [204]. Compared to CD4^+^ T cells, macrophages are more resistant to HIV cytopathic effects and survive longer with the infection; thus, they have an increased likelihood of interacting with and infecting uninfected cells [205]. Macrophages tend to reside in secondary lymphoid organs, which, due to their design, have reduced penetration of antiretroviral drugs; thus, viral replication may be active even in the presence of antiretroviral therapy (ART) [206,207]. Finally, unlike infected CD4^+^ T cells, infected macrophages have virus-filled membrane-connected compartments that appear to aid the release of viruses [208,209]. 

### 5.4. CD4^+^ T Cells

CD4^+^ T cells are crucial components of the immune system and play a key role in mounting an effective response against viruses such as HIV [9]. However, HIV specifically targets and infects CD4^+^ T cells, leading to a gradual depletion of this cell population and ultimately resulting in the onset of AIDS [81].

The mechanisms underlying the CD4^+^ T cell response to HIV infection involve a variety of signaling pathways and biochemical interactions [210]. Upon the initial encounter with HIV, CD4^+^ T cells become activated and initiate a series of intracellular signaling events, including calcium flux and protein kinase C activation [136,211,212]. These signaling pathways culminate in the mobilization of transcription factors, such as NF-κB, which promote the expression of pro-inflammatory cytokines and chemokines that recruit and activate other immune cells [213].

### 5.5. CD8^+^ T Cells 

CD8^+^ T cells recognize and directly eliminate virus-infected cells. In the acute phase of HIV infection, there is an increase in CD8^+^ T cell activity due to APCs and CD4 T cell stimulation, resulting in CD8^+^ T cells killing virus-infected cells by releasing granzymes, which can induce apoptosis in the target cell and a pore-forming protein called perforin, which perforates the cell membrane of the target cell, thereby killing the cell [214]. 

CD8^+^ T cells recognize infected cells through the presentation of viral peptides on major histocompatibility MHC 1 molecules [215]. Once they encounter an antigen on MHC 1 molecules, they become activated, gain cytotoxic activity and additionally secrete a variety of cytokines including IFN-γ, which inhibit viral replication and create an antiviral environment [79,216].

When activated, CD8^+^ T cells can produce cytokines such as IFN-γ that enhance the cytotoxic activity of the CD8^+^ T cell and activate other immune cells, such as macrophages [217]. IFN-γ is produced by the CD8^+^ T cell following activation and can directly inhibit viral replication by inducing antiviral activity in infected cells [218]. 

In addition, CD8^+^ T cells can differentiate into different subsets, such as effector and memory T cells, which differ in their functions and responsiveness to signals [219]. Effector T cells are short-lived and have potent cytotoxic activity against target cells [220]. Memory T cells can persist for years and mount a rapid and enhanced response upon re-exposure to an antigen [221]. In early HIV infection, the production of an effector CD8^+^ T cell is crucial for the control of viral replication, while long-lived memory CD8^+^ T cells are essential for the resolution of the infection [215,222]. However, in chronic infection, the CD8^+^ T cell functionality becomes progressively impaired, contributing to the failure of the immune system [223]. The CD8^+^ T cell impairment in chronic HIV could be driven by a variety of factors, including CD4^+^ T cell depletion, CD8^+^ T cell exhaustion, the expansion of dysfunctional CD8^+^ T cell subsets and the expansion of Tregs that suppress CD8^+^ T cell activity [9,223,224].

CD8^+^ T cell exhaustion is characterized by the loss of effector functions and the upregulation of inhibitory receptors such as the programmed cell death-1 (PD-1) and T cell immunoglobulin and mucin domain-containing protein 3, which inhibit T cell activation and proliferation [12,225]. Chronic stimulation by persistent viral antigen is a major driver of CD8^+^ T cell exhaustion in chronic HIV infection [226]. The dysfunctional CD8^+^ T cell l subsets that accumulate in chronic HIV infection include terminally differentiated CD8^+^ T cells and CD8^+^ T cells with reduced cytokine production, and these, while physically present, have little or no impact on the infection [226]. The accumulation of Tregs, partially driven by the DC immune regulatory enzyme, indoleamine 2,3 dioxygenase enzyme activity, suppresses immune CD8^+^ T cell immune responses [227,228]. 

## 6. Influence of Sex on HIV Transmission and Immune Responses

There are notable sex differences in HIV infection transmission and progression. HIV infection in females is marked by a stronger initial immune response, characterized by a high CD4^+^ T cell count, low viral load and high CD8^+^ T cell activity, while infection in males is marked by high viral load, a lower CD4^+^ T cell count and low CD8^+^ T cell cytotoxic activity [229,230]. However, there is early immune exhaustion in females, accelerating the progression to AIDS at a rate comparable to that of males, and progression to AIDS occurs at a lower viral load compared to that of males [231]. 

Both the foreskin and the vaginal mucosa contain CD4^+^ T cells, which can be infected by HIV during sexual intercourse with an infected person; however, male CD4^+^ T cells express higher CCR5 receptors when compared to females, which implies that males are more likely to be infected in one sexual encounter with higher viral particles, thus partially explaining the higher viral loads in males in primary HIV infection [232,233]. Furthermore, the Langerhans cells in the vagina and foreskin transport viral particles to the local lymphatics, where they present the viruses to the CD4^+^ T cells in the process of infecting them and spreading the infection [234]. Therefore, males who undergo circumcision have up to 60% reduced chances of contracting HIV because of the loss of the foreskin, which significantly reduces both CD4^+^ T cells and Langerhans cells [235,236,237]. The female genital tract mucosal membrane structure, cells and microbiota are influenced by hormones that regulate the menstrual cycle [238,239]. Estrogen thickens the mucosal membranes and enhances stronger immune responses against infection; thus, during the menstrual phases of estrogen influence, women are at a lower risk of HIV infection compared to the periods under progesterone influence [240,241,242]. Female genital tract mucosal damage significantly increases the likelihood of acquiring HIV infection during sexual intercourse; hence, a thicker and stronger mucosal membrane is important in HIV prevention [243]. 

It is a well-established fact that females generally mount stronger immune responses to both self and non-self-antigens, including viral infection: therefore, females are more prone to autoimmune disease than males [244]. While the mechanisms behind the sex differences are not fully understood, several genetic and physiological mechanisms are thought to be responsible for the stronger immune responses mounted by females compared to males in response to HIV infection and for how this may influence disease progression [245].

The first mechanism that can explain the stronger immune responses in females is the presence of X chromosome-linked genes that contribute to the higher expression of TLRs in females [246]. TLR7, which is higher in females, is located on the X chromosome, and females have two copies of the X chromosome, as opposed to males, who only have one [247]. For one copy of the X chromosome, it is proven that up to 23% of the genes escape inactivation; thus, females potentially have higher levels of TLR7 due to gene dosage effects [248]. The male Y chromosome carries very few genes, which mainly are involved in sex determination; thus, males possess no gene dosage advantage like females do, thus leading to weaker immune activity [249,250].

The differences in immune response between sexes could also be attributed to sex hormones. Sex hormones have been shown to regulate the expression of TLRs, particularly TLR7 and TLR8, which recognize single-stranded RNA viruses such as HIV; this can lead to a stronger innate immune response against HIV and may help to control viral replication [251,252,253]. Several studies have shown that estrogen can upregulate TLR expression in immune cells, which could contribute to the higher expression of TLR in females [254,255,256]. On the other hand, testosterone has been shown to downregulate TLR expression, which could contribute to lower TLR expression in males [257,258]. 

Estrogen has also been shown to stimulate immune response by increasing the number and activity of immune cells such as T cells and B cells, and it achieves this by stimulating the secretion of cytokines from a variety of cells, including immune cells such as monocytes, macrophages, dendritic cells and T cells [259,260,261]. Estrogen modulates the production of cytokines such as interleukin-1 (IL-1), interleukin-6 (IL-6), TNF-alpha and IFN-γ, among others [262,263,264,265,266,267]. These cytokines are important in regulating immune responses and play a role in inflammatory and autoimmune diseases [268,269,270,271]. Estrogen stimulates cytokine release by binding to the Estrogen receptors (ERs), ER-alpha (ERα) and ER-beta (ERβ), which are expressed on the surface of immune cells [272]. Once activated by estrogen binding, these receptors translocate to the nucleus and bind to estrogen response elements (ERE) on the DNA, leading to the transcription of target genes that can regulate cytokine production [273]. Overall, these processes will result in the proliferation and activation of T cells by the activity of IL-2, TNF-alpha and IFN-γ, B cell proliferation and differentiation through IL-6 activity, DC maturation and activation by TNF-alpha activity and macrophage activation via TNF-alpha and IL-1 activity [255,259,274]. 

Estrogen can also bind to membrane-associated estrogen receptors (mERs), such as G protein-coupled receptor 30 (GPR30), which activate intracellular signaling cascades, such as the mitogen-activated protein kinase (MAPK) pathway [275,276,277]. These signaling pathways regulate cytokine production by immune cells [278,279]. The MAPK pathway can activate the c-Jun N-terminal kinase (JNK), extracellular signal-regulated kinase (ERK) and p38 MAPK sub-pathways, which regulate different aspects of immune cell activation [279]. For example, the JNK pathway activates the transcription factor AP-1, which can regulate cytokine gene expression: several cytokines, including IL-1, IL-2 and IFN-gamma, are activated by AP-1 [280,281,282]. The ERK pathway activates the transcription factor Elk-1, which regulates the genes involved in proliferation and survival [283,284]. The p38 MAPK pathway can also regulate cytokine gene expression and is involved in the activation of innate immune responses; therefore, the net effect of estrogen activity is a stronger immune response to infection [285].

To demonstrate the activity of estrogen, post-menopausal women living with HIV (WLH) have weaker immune responses to HIV and a higher viral load when compared to pre-menopausal WLH: therefore, estrogen is key to mounting stronger immune responses in females [286,287].

On the other hand, androgens inhibit the activation of T cells by reducing the expression of key signaling molecules, such as CD28 and CD4, which are necessary for T cell activation and also inhibit the production of interleukin-2 (IL-2), which is important for T cell proliferation and survival [288]. Androgens can modulate cytokine production by immune cells [258]. For example, they reduce the production of pro-inflammatory cytokines such as IL-1, IL-6 and TNF-alpha and increase the production of anti-inflammatory cytokines such as interleukin-10 (IL-10). 

Furthermore, androgens promote the development of Tregs, which suppress immune responses and enhance the expression of molecules that promote Treg development, such as transforming growth factor-beta (TGF-beta) and interleukin-10 (IL-10) [289,290]. Androgens can inhibit the function of dendritic cells, which are important for initiating immune responses by reducing the expression of molecules that are necessary for dendritic cell maturation and function, such as CD80, CD86 and MHC class II molecules [291,292,293]. Finally, androgens also suppress natural killer (NK) cell activity by reducing the expression of activating receptors on the surface of NK cells, such as NKG2D and NKp46; the net effect is the weaker immune response seen in males [293,294,295].

Overall, the gender-related dynamics of HIV immunopathology may have implications for the management of HIV, which may include the reduced bioavailability of active forms of the drugs to hormonal or other biological influences [296].

## 7. Strengths and Limitations

The strength of our review is that we have highlighted several key issues that influence the HIV–host cell interactions which include the role of viral products in manipulating the host cell responses, how the host immune system responds to the infection and further coupling the former with sex differences in HIV-induced immunopathology. 

The limitations are as follows: we did not investigate the influence of race, genetics (non-sex-related), coinfection and environmental factors. All these factors are important in the disease outcome. The HIV host cell interactions are complex and thus often difficult to understand; however, understanding the HIV–host cell interactions is key to the development of an effective cure for HIV infection [297,298].

## 8. Conclusions 

The pathogenesis of HIV is determined by an intricate interaction between the host and the virus. HIV has evolved to escape immune surveillance, infect human cells and continue to replicate within host cells through several complex mechanisms such as the downregulation of MHC molecules, the production of non-neutralizing antibodies and other HIV-related proteins to induce immune exhaustion, the activation of apoptosis and the dysregulation of the JAK/STAT pathway. Host defense mechanisms promote control and decreased HIV viral replication through the downstream activation of several transduction pathways that serve to eliminate the virus and infected cells. Sex differences are important in both infection and HIV disease progression, with females being more vulnerable to infection while on the other hand being capable of mounting a stronger immune response to the infection compared to men. 

For future studies, it may be of value to thoroughly investigate how race, genetics and environmental factors (such as climate) influence host–cell interactions. This would be of great importance in improving HIV disease management and finding a cure. For example, since the monocyte has been identified to be a key driver of latency, medications are being developed and modeled to target viruses housed in macrophages [299,300].

## Data Availability

All data are contained within the manuscript.
